# Brain PET in the era of anti-amyloid-β antibody therapy for Alzheimer disease

**DOI:** 10.1007/s11604-026-01998-4

**Published:** 2026-04-25

**Authors:** Kazunari Ishii

**Affiliations:** Department of Radiology, Kobe University Graduate School of Medicine, 7-5-2 Kusunoki-Cho, Chuo-Ku, Kobe, Hyogo, 650-0017 Japan

**Keywords:** Alzheimer’s disease, Amyloid PET, Tau PET, Anti-amyloid-β antibody, Lecanemab, Donanemab

## Abstract

Here the current and emerging roles of brain positron emission tomography (PET) in Alzheimer’s disease (AD) in the era of anti-amyloid-β antibody therapy, with a focus on clinical applications, methodological considerations, and future perspectives were reviewed. A narrative review of the literature on PET imaging in AD, including FDG-PET, amyloid PET, and tau PET, was conducted with particular emphasis on their clinical utility in diagnosis, and disease monitoring. Relevant guidelines, including appropriate use criteria and Japanese clinical guidelines, were also reviewed. FDG-PET provides valuable information for the differential diagnosis of neurodegenerative dementias based on characteristic hypometabolic patterns, although its role remains supportive due to the lack of direct assessment of molecular pathology. Amyloid PET enables noninvasive visualization of cerebral amyloid-β deposition and has become essential for confirming eligibility for anti-amyloid therapies. Standardized use criteria and interpretation guidelines are critical for appropriate clinical implementation. Quantitative approaches, such as standardized uptake value ratios (SUVRs) and the Centiloid scale, improve comparability across studies and institutions. Tau PET reflects neurofibrillary pathology and correlates with disease severity and progression, with increasing relevance for patient stratification. In addition, recent advances in high-resolution dedicated brain PET systems and artificial intelligence–based image analysis are expected to enhance diagnostic performance and workflow efficiency. In the era of disease-modifying therapy, brain PET imaging has become integral to the clinical management of AD. Amyloid PET is indispensable for treatment eligibility, while tau PET provides complementary information on disease stage and prognosis. Ongoing technological and methodological advancements will further expand the role of PET imaging in precision medicine for dementia.

## Introduction

Alzheimer’s disease (AD) is the most common cause of dementia and is neuropathologically characterized by extracellular amyloid-β (Aβ) plaques and intracellular neurofibrillary tangles composed of hyperphosphorylated tau protein. For decades, the diagnosis of AD has relied largely on clinical symptoms, supported by structural and functional neuroimaging findings. However, the recent approval of anti-amyloid-β monoclonal antibodies, such as lecanemab [1] and donanemab [2], has fundamentally transformed AD from a disorder managed primarily with symptomatic therapies into one that is amenable to disease-modifying treatment.

This paradigm shift has markedly increased the importance of in vivo biomarkers that enable direct visualization of AD pathology. Positron emission tomography (PET) allows noninvasive assessment of cerebral glucose metabolism, Aβ deposition, and tau accumulation, thereby providing complementary information across the disease continuum. In particular, amyloid PET has become essential for confirming amyloid positivity prior to the initiation of anti-Aβ antibody therapy, whereas tau PET is emerging as a promising biomarker for disease staging and prognostication [3].

In this review, the evolving role of brain PET imaging in the era of anti-Aβ antibody therapy, with a focus on clinical applications, methodological considerations, and future perspectives are discussed.

## FDG-PET

^18^F-FDG PET reflects neuronal glucose metabolism and synaptic activity. Characteristic hypometabolic patterns are well established for AD [4], dementia with Lewy bodies, and frontotemporal lobar degeneration [5], making FDG-PET a valuable tool for differential diagnosis [6, 7]. However, FDG-PET does not directly visualize the underlying molecular pathology and therefore plays a supportive rather than definitive role in the era of disease-modifying therapies.

In the United States, FDG-PET has been covered by health insurance for the differential diagnosis between AD and frontotemporal dementia (FTD) since 2004 [8]. In contrast, in Japan, it has not yet been reimbursed for the differential diagnosis of dementia. Evidence supporting its clinical utility for reimbursement approval is currently being generated in Japan [9].

## Amyloid PET: A Cornerstone in Anti-Aβ Antibody Therapy

Amyloid and tau PET enable direct visualization of AD pathology in vivo. Their clinical relevance has increased substantially with the introduction of anti-Aβ antibody therapies, shifting PET imaging from a research tool to a central component of routine dementia care [3].

### Clinical significance

Amyloid PET is the only imaging modality capable of noninvasively visualizing cerebral Aβ deposition. In the current therapeutic landscape, amyloid positivity is a prerequisite for treatment with anti-Aβ antibodies. Conversely, a negative amyloid PET scan effectively excludes AD as the primary cause of cognitive impairment, reflecting its high negative predictive value.

### Comparison between CSF biomarkers and PET imaging

Cerebrospinal fluid (CSF) biomarkers and PET imaging represent two complementary approaches for assessing AD pathology. CSF analysis enables sensitive detection of soluble biomarkers, including decreased amyloid-β42 (Aβ42) and increased total tau (t-tau) and phosphorylated tau (p-tau), thereby reflecting early molecular changes in the disease process [10]. In contrast, PET imaging allows in vivo visualization of fibrillar amyloid and tau deposition, facilitating the evaluation of the regional distribution and topography of pathological changes. While CSF biomarkers are generally more accessible, cost-effective, and sensitive to early alterations, PET imaging provides superior spatial information and is particularly valuable for disease staging and differential diagnosis. In clinical practice, these modalities are not mutually exclusive but rather complementary, and are especially useful in atypical cases and in determining eligibility for disease-modifying therapies. Recently, blood-based biomarkers have also been increasingly investigated and are expected to play an important role as screening tools in the future.

### Clinically available PET tracers

**Fig. 1:**
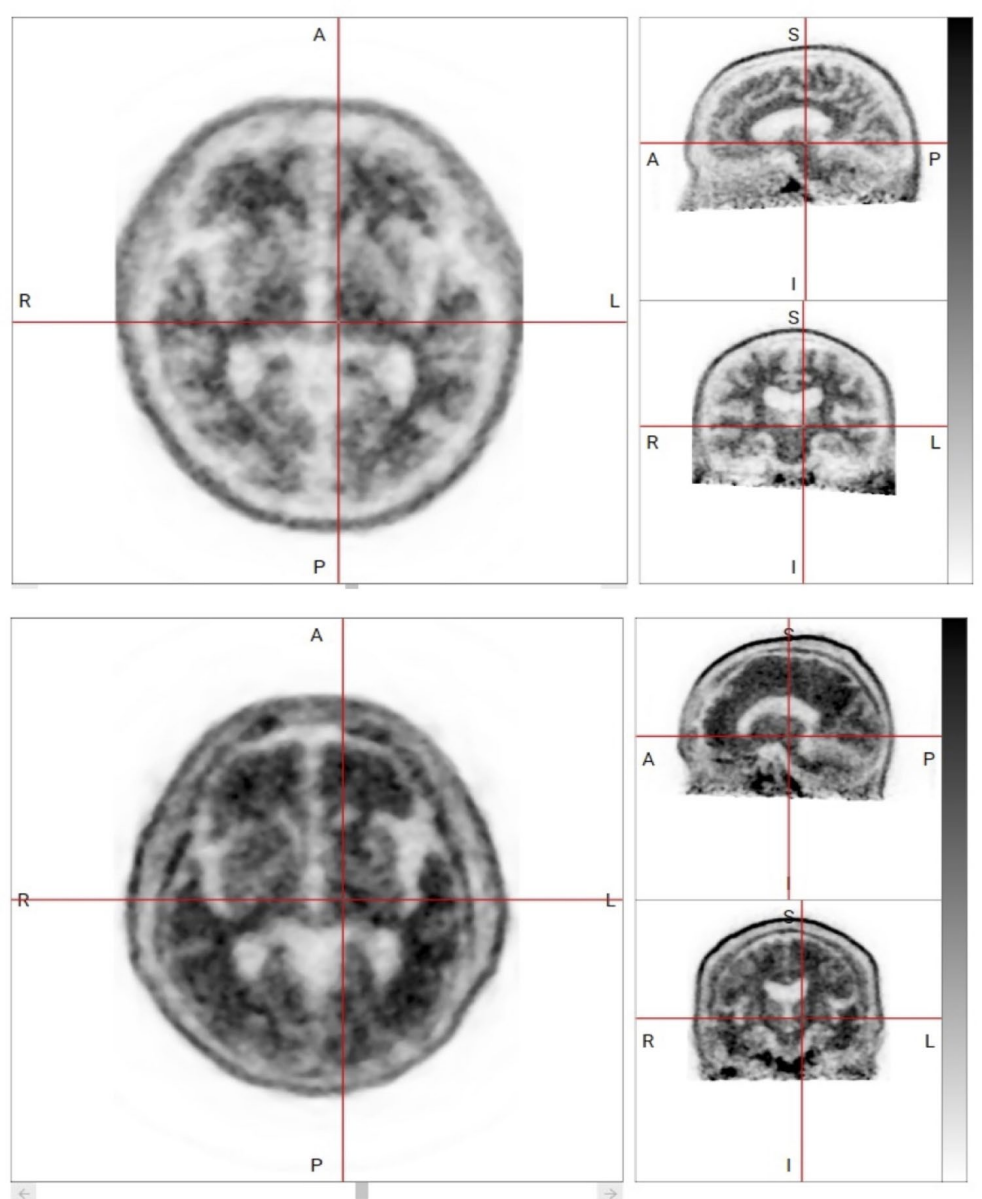
^18^F-florbetapir amyloid PET images **A** Amyloid-negative case. No significant increase in tracer uptake is observed in the gray matter, aside from nonspecific uptake in the white matter (CL =  − 4). **B** Amyloid-positive case. In addition to nonspecific uptake in the white matter, significantly increased uptake is observed in the gray matter of the frontal lobe, temporal lobe, posterior cingulate/precuneus, and parietal lobe (CL = 65)

**Fig. 2:**
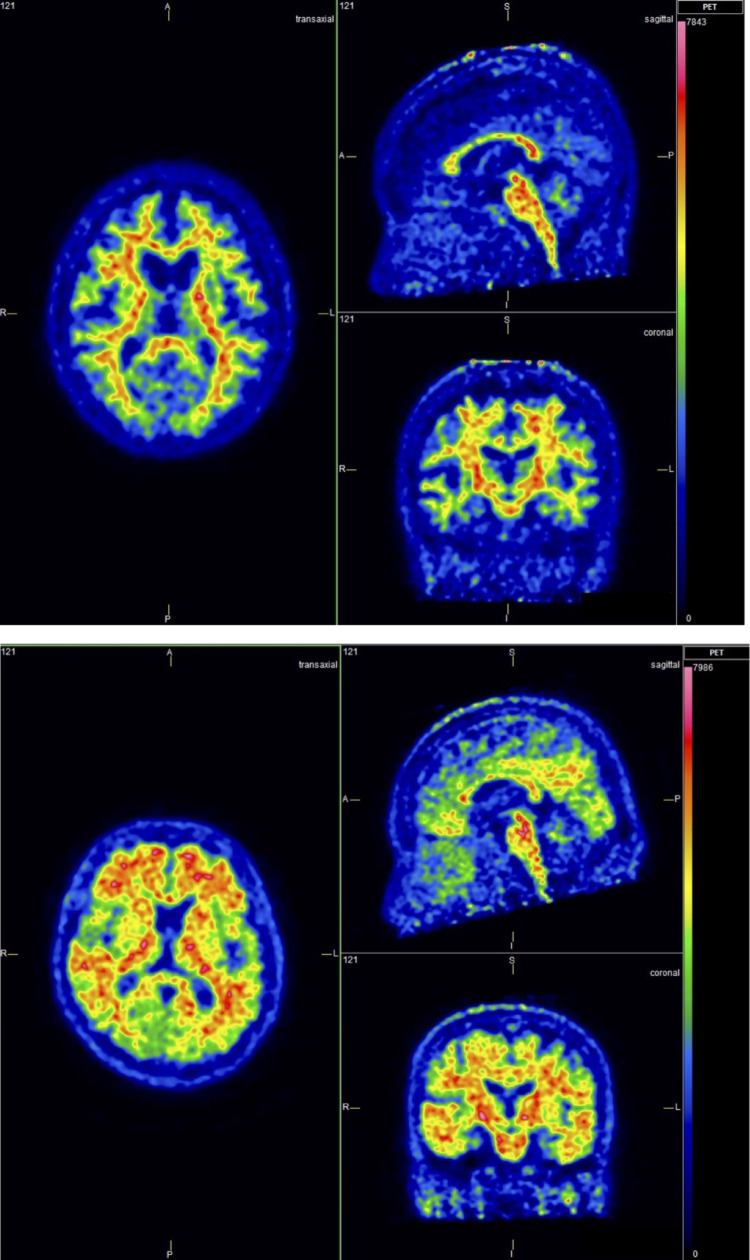
^18^F-flutemetamol amyloid PET images. **A** Amyloid-negative case. No significant increase in tracer uptake is observed in the gray matter, aside from nonspecific uptake in the white matter (CL =  − 4). **B** Amyloid-positive case. Significantly increased uptake is observed in the gray matter of the frontal lobe, posterior cingulate/precuneus, lateral temporal lobe, and parietal lobe, as well as in the striatum, in addition to nonspecific uptake in the white matter (CL = 83)

The first amyloid PET tracer developed was ^11^C-Pittsburgh compound B (PiB) [11]. This tracer has demonstrated excellent performance as an amyloid PET radiopharmaceutical and is still regarded as the gold standard for amyloid PET imaging. However, because it is labeled with ^11^C, which has a short half-life of approximately 20 min, its use is limited to facilities equipped with a cyclotron and automated synthesis systems. Furthermore, the developer, the University of Pittsburgh, has restricted its use to research purposes only. Consequently, ^11^C-PiB is not available for routine clinical practice. Currently approved ^18^F-labeled amyloid PET tracers include:^18^F-florbetapir [12] (Fig. 1), ^18^F-flutemetamol [13] (Fig. 2), and ^18^F-florbetaben [14]. Although these tracers differ slightly in pharmacokinetics and recommended acquisition protocols, all provide robust visualization of cortical amyloid deposition when interpreted according to tracer-specific guidelines. ^18^F-NAV4694 has also been developed and shows a strong correlation with the uptake distribution of ^11^C-PiB [15] with high specificity for Aβ deposition [16]; however, it has not yet been approved by regulatory authorities.

### Appropriate use criteria

Amyloid PET is most appropriate in patients with mild cognitive impairment (MCI) or mild dementia in whom AD is suspected and disease-modifying therapy is being considered. In contrast, amyloid PET is not recommended for screening asymptomatic individuals or for confirming diagnosis in patients with advanced dementia, where therapeutic implications are limited [17, 18].

The Japanese Guidelines for the Appropriate Use of Amyloid PET aim to promote the proper production and clinical use of amyloid PET tracers in dementia care. The fourth revised edition of the guidelines has been issued under the supervision of seven academic societies: the Japanese Society of Nuclear Medicine, Japan Society for Dementia Research, Japanese Society of Neurology, Japanese Society of Neurological Therapeutics, Japanese Society of Psychiatry and Neurology, Japan Geriatrics Society, and Japanese Psychogeriatric Society. The guidelines (in Japanese) can be downloaded from the website of the Japanese Society of Nuclear Medicine (JSNM) [https://jsnm.org/archives/655/]. The main points of the guidelines are summarized below.

### Appropriateness of PET examination ordering

Amyloid PET examinations should be requested by physicians with sufficient knowledge and experience in dementia, after appropriate clinical evaluation of the patient. Ideally, such examinations should be ordered by physicians with a high level of expertise in dementia diagnosis and management. Therefore, physicians who request amyloid PET examinations are required to complete designated training programs. When amyloid PET is used in the context of treatment with lecanemab or donanemab, physicians must also meet the requirements specified in the “Guidelines for the Proper Use of Lecanemab or Donanemab (recombinant)”.

Appropriate and inappropriate use are defined as follows in the guidelines.

### Appropriate use

- Patients with MCI or dementia with atypical clinical presentations, in whom a definitive diagnosis is required to guide appropriate treatment.
- Patients with MCI or dementia with an atypical age at onset (onset before 65 years), in whom a definitive diagnosis is required to guide appropriate treatment.
- Patients with MCI or mild AD patients in whom confirmation of the diagnosis of AD is required to determine eligibility for anti-Aβ antibody therapy.
- Confirmation of Aβ plaque removal following treatment with anti-Aβ antibody therapy, for the purpose of deciding treatment discontinuation, continuation, or re-initiation.

### Inappropriate use

- Patients with advanced, severe dementia.
- Cases in which clinical symptoms and disease course are typical and the clinical diagnosis of AD is clear, and the purpose of testing is only to confirm the diagnosis rather than to determine eligibility for disease-modifying therapy.
- Preclinical diagnosis of AD in asymptomatic individuals.
- Use based solely on the presence of a family history of dementia or carriage of the apolipoprotein E (APOE) ε4 allele.
- Individuals who complain of subjective memory problems but show no objective evidence of cognitive impairment.
- Assessment of disease severity in AD.
- Non-medical purposes, such as health check, employment screening or insurance-related evaluations.
- Situations in which adequate consideration cannot be given to the potential psychological or social impact of the test results.

### Appropriateness of imaging facilities

#### Production of amyloid PET tracers

When amyloid PET tracers are synthesized within a medical institution, the synthesis process must comply with the manufacturer’s instructions for the synthesis system, package inserts, and the standards established by the JSNM. Appropriate synthesis environments, operation of equipment, and quality control procedures are required. Because amyloid PET tracers have a short half-life and are administered before the results of sterility testing become available, strict assurance of sterility is particularly important. To maintain consistent quality across different institutions, ensuring the reliability of quality testing procedures is also required.

#### Requirements for imaging facilities

Amyloid PET examinations should be performed at medical institutions that have appropriate infrastructure and expertise in nuclear medicine and dementia diagnosis. Imaging facilities are required to ensure proper handling, storage, and administration of amyloid PET tracers in accordance with the standards defined by the JSNM.

Furthermore, facilities must establish appropriate protocols for patient selection, radiotracer administration, image acquisition, and safety management. Close collaboration between nuclear medicine physicians, neurologists, psychiatrists, and dementia specialists is encouraged to ensure appropriate clinical utilization of amyloid PET imaging.

#### Interpretation of amyloid PET images

Amyloid PET images should be interpreted by physicians with sufficient training and experience in nuclear medicine imaging and dementia diagnosis. To maintain diagnostic accuracy and consistency, physicians interpreting amyloid PET images are required to complete specific training programs that include standardized interpretation criteria and case-based learning. The interpretation criteria for each tracer are presented in Table 1.

**Table 1:** Standardized interpretation of amyloid PET tracers

Tracer	Injected activity and acquisition	Display method	Image evaluation	Criteria for positivity
^18^F-florbetapir (Amyvid®)	370 MBq ± 10%; 30–50 min post-injection (standard 50 min); 10–20 min acquisition	Grayscale (white background)	Axial images (inferior to superior); adjust to visualize gray–white matter boundary; evaluate frontal, parietal, temporal, and occipital lobes using cerebellum as reference	Reduced or absent gray–white matter contrast in ≥ 2 regions, or cortical gray matter uptake greater than adjacent white matter in ≥ 1 region
^18^F-flutemetamol (Vizamyl®)	185 MBq ± 10%; 60–120 min post-injection (standard 90 min); 20–30 min acquisition	Color scale (Rainbow or Sokoloff)	Axial, coronal, and sagittal planes aligned to AC–PC line; normalize intensity to pons/cerebellar peduncles; evaluate frontal, posterior cingulate/precuneus, lateral temporal, parietal lobes, and striatum	Loss of normal sulcal white-matter pattern or increased cortical gray matter uptake in ≥ 1 region
^18^F-florbetaben (Neuraceq®)	300 MBq ± 20%; 45–130 min post-injection (standard 90 min); 20 min acquisition	Grayscale (black background; axial only)	Axial images only; evaluate frontal, lateral temporal, posterior cingulate/precuneus, and parietal lobes using cerebellum as reference	Cortical gray matter uptake equal to or greater than white matter in the majority of slices in ≥ 1 region

In addition, institutions are encouraged to implement quality assurance measures such as peer review and multidisciplinary case discussions. These measures aim to reduce inter-reader variability and ensure reliable interpretation of amyloid PET findings in clinical practice.

The Japanese guidelines share several fundamental principles with the Appropriate Use Criteria (AUC) proposed by the Alzheimer’s Association and the Society of Nuclear Medicine and Molecular Imaging (SNMMI) in the United States and with the recommendations issued by the European Association of Nuclear Medicine (EANM) [17–19].

The AUC framework primarily focuses on defining clinical scenarios in which amyloid PET is appropriate, uncertain, or rarely appropriate, such as unexplained MCI, atypical or mixed dementia presentations, and early-onset dementia. In contrast, the Japanese guidelines place stronger emphasis on regulating the clinical implementation process, including physician training requirements, facility qualifications, and appropriate radiopharmaceutical production and quality control.

Another distinctive feature of the Japanese system is the integration of amyloid PET into national health insurance reimbursement policies, particularly in relation to eligibility assessment for anti-Aβ antibody therapies such as lecanemab and donanemab.

### Visual interpretation and pitfalls

**Fig. 3:**
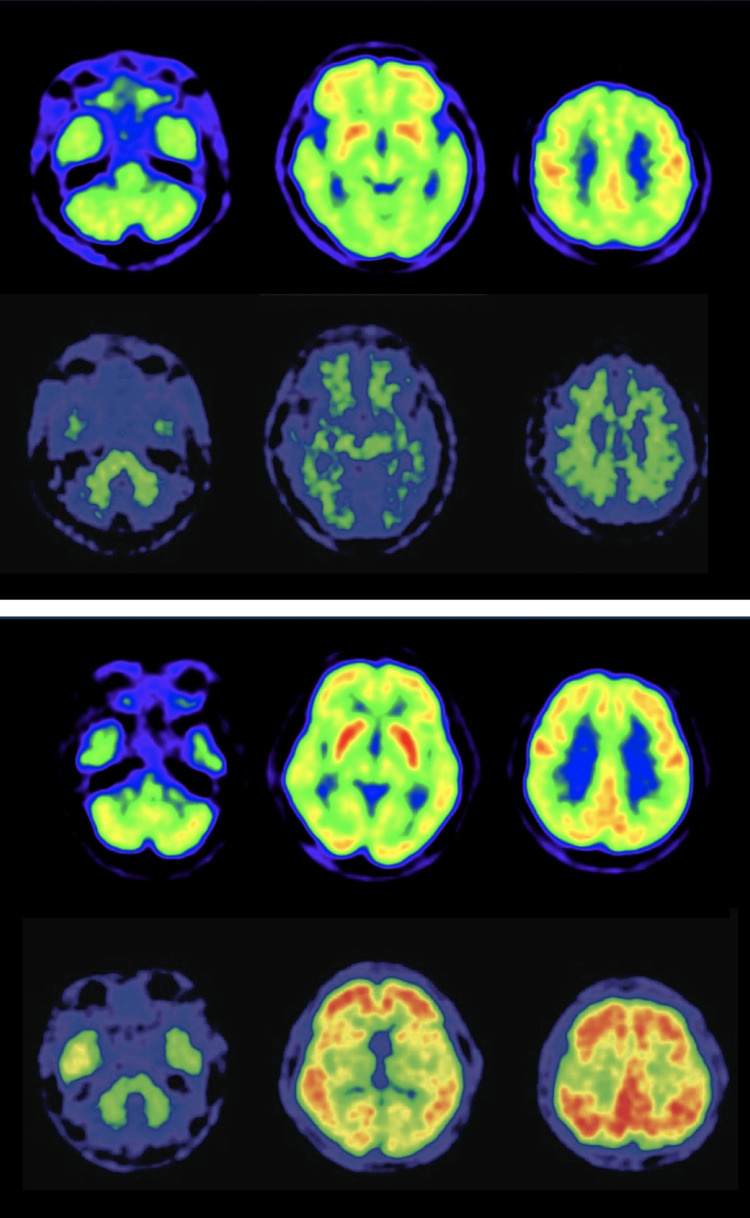
**A** and **B**. Amyloid-negative DLB case **A** and amyloid-positive DLB case **B**: Upper row: ^18^F-FDG PET images of glucose metabolism; lower row: amyloid PET images using ^11^C-PiB. **A** Diffuse reduction of cerebral glucose metabolism, including the occipital lobes, is observed, sparing the primary sensorimotor cortex, striatum, and mid-cingulate cortex. No significant increase in tracer uptake is observed in the cortical gray matter on amyloid PET. **B** Diffuse reduction of cerebral glucose metabolism, including the occipital lobes, is observed, sparing the primary sensorimotor cortex, striatum, and mid-cingulate cortex. On amyloid PET, increased uptake is observed in the gray matter of the frontal lobe, lateral temporal lobe, posterior cingulate/precuneus, and parietal lobe. Mild uptake is also observed in the striatum

Clinical interpretation is currently based on binary visual assessment (positive vs negative). Over-interpretation of focal or nonspecific uptake should be avoided, as false-positive findings may lead to inappropriate treatment decisions. Given that amyloid PET results directly influence eligibility for therapy, standardized training and strict adherence to reading criteria are essential. Approximately half of patients with DLB are amyloid PET–positive [20]; therefore, amyloid positivity alone should not be interpreted as indicative of AD, and careful interpretation is required (Fig. 3A and B).

### Quantitative assessment: SUVR and the centiloid scale

Semi-quantitative analysis using standardized uptake value ratios (SUVRs) is widely applied in both research and clinical trials. The Centiloid scale further facilitates harmonization across tracers, scanners, and institutions by expressing amyloid burden on a standardized 0–100 scale [11]. Recently, a novel method for amyloid PET evaluation has been proposed based on a gray matter–specific histogram using a three-component model, which differs fundamentally from conventional approaches such as SUVR and the Centiloid scale [21].

Despite these advantages, quantitative measures are influenced by factors such as acquisition timing, reconstruction algorithms, and reference region selection [22–25]. Therefore, quantitative results should be interpreted in conjunction with visual assessment and clinical context.

## Tau PET: linking pathology to clinical phenotype

### Biological relevance

Tau accumulation correlates strongly with neuronal loss, cognitive impairment, and disease progression. Accordingly, tau PET provides critical information on disease stage and prognosis, complementing amyloid PET.

### First-generation tau tracers

First-generation tau PET tracers, such as ^18^F-AV-1451 [26], ^18^F-THK5351 [27] and ^11^C-PBB3 [28], have been associated with off-target binding to structures other than tau deposits. However, among these tracers,^18^F-AV-1451 is currently the only one approved by the Food and Drug Administration (FDA) [29].

### Second-generation tau tracers

**Fig. 4:**
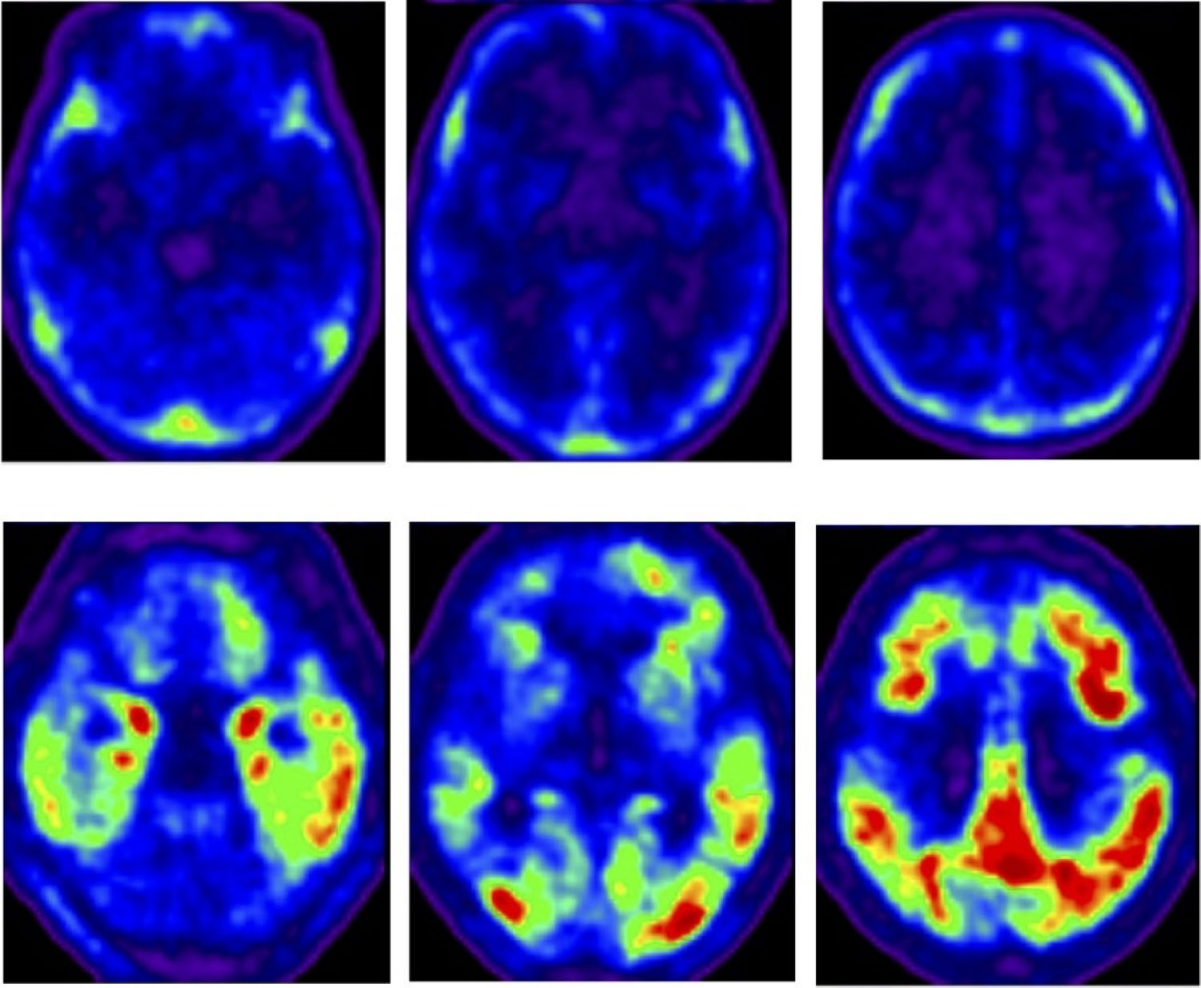
^18^F-MK-6240 tau PET images. Upper row: Tau-negative non–AD case. Nonspecific uptake is observed in the meninges and partially in the venous sinuses, with no significant increase in tracer uptake in the cerebral cortical gray matter. Lower row: Tau-positive AD case. Markedly increased uptake is observed beginning in the medial temporal lobe and extending to the lateral temporal lobe, frontal lobe, parietal cortex, and posterior cingulate/precuneus

**Fig. 5:**
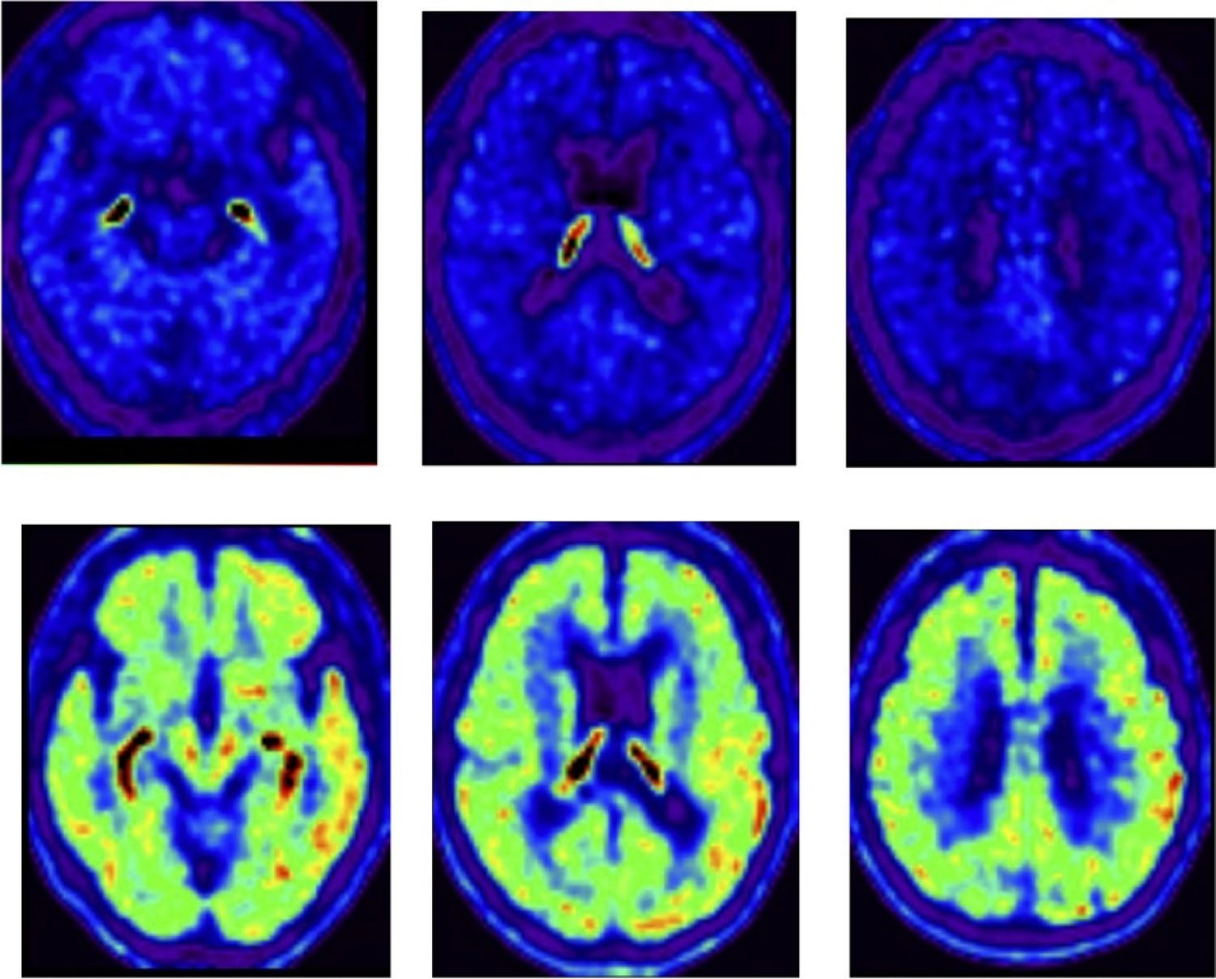
^18^F-PM-PBB3 tau PET images. Upper row: Tau-negative non–AD case. Nonspecific uptake is observed in the choroid plexus, with no significant increase in tracer uptake in the cerebral cortical gray matter. Lower row: Tau-positive AD case. In addition to nonspecific uptake in the choroid plexus, diffuse increased uptake is observed in the cerebral cortical gray matter, with slightly greater uptake in the left temporo-occipital region

Second-generation tau tracers, including ^18^F-MK-6240 [30] (Fig. 4) and ^18^F-PM-PBB3 [31] (Fig. 5), demonstrate improved specificity and reduced off-target binding compared with first-generation tracers. ^18^F-MK-6240, in particular, shows high affinity for AD-type tau (3R + 4R) and enables high-contrast visualization of tau distribution consistent with Braak staging.

### Implications for anti-Aβ antibody therapy

Recent studies suggest that baseline tau burden influences the therapeutic response to anti-Aβ antibody therapy. Patients with lower tau accumulation appear to derive greater clinical benefit, highlighting the potential role of tau PET in patient stratification and outcome prediction [32].

## Technological advances: high-resolution PET and AI

**Fig. 6:**
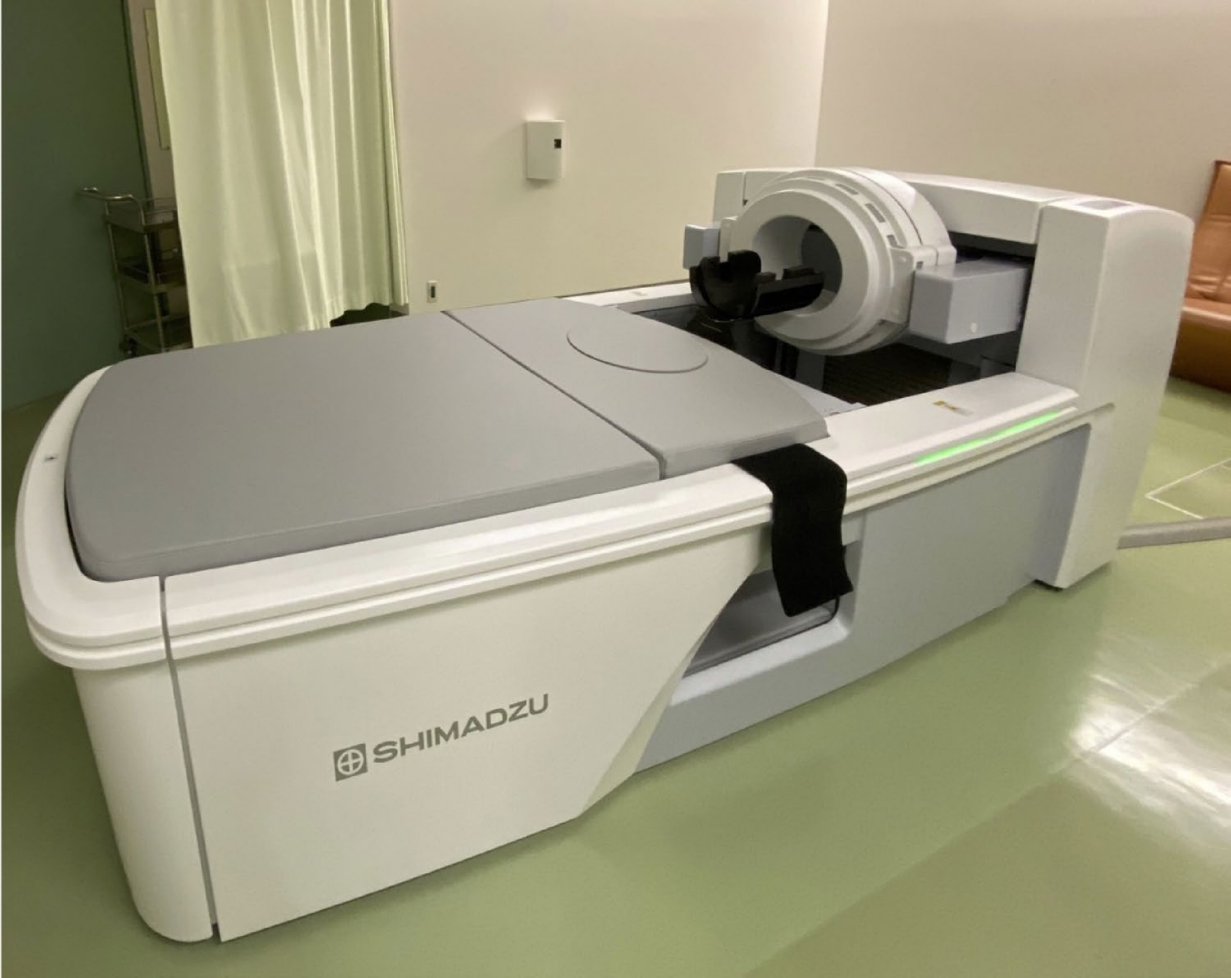
Appearance of dedicated brain PET scanner

**Fig. 7:**
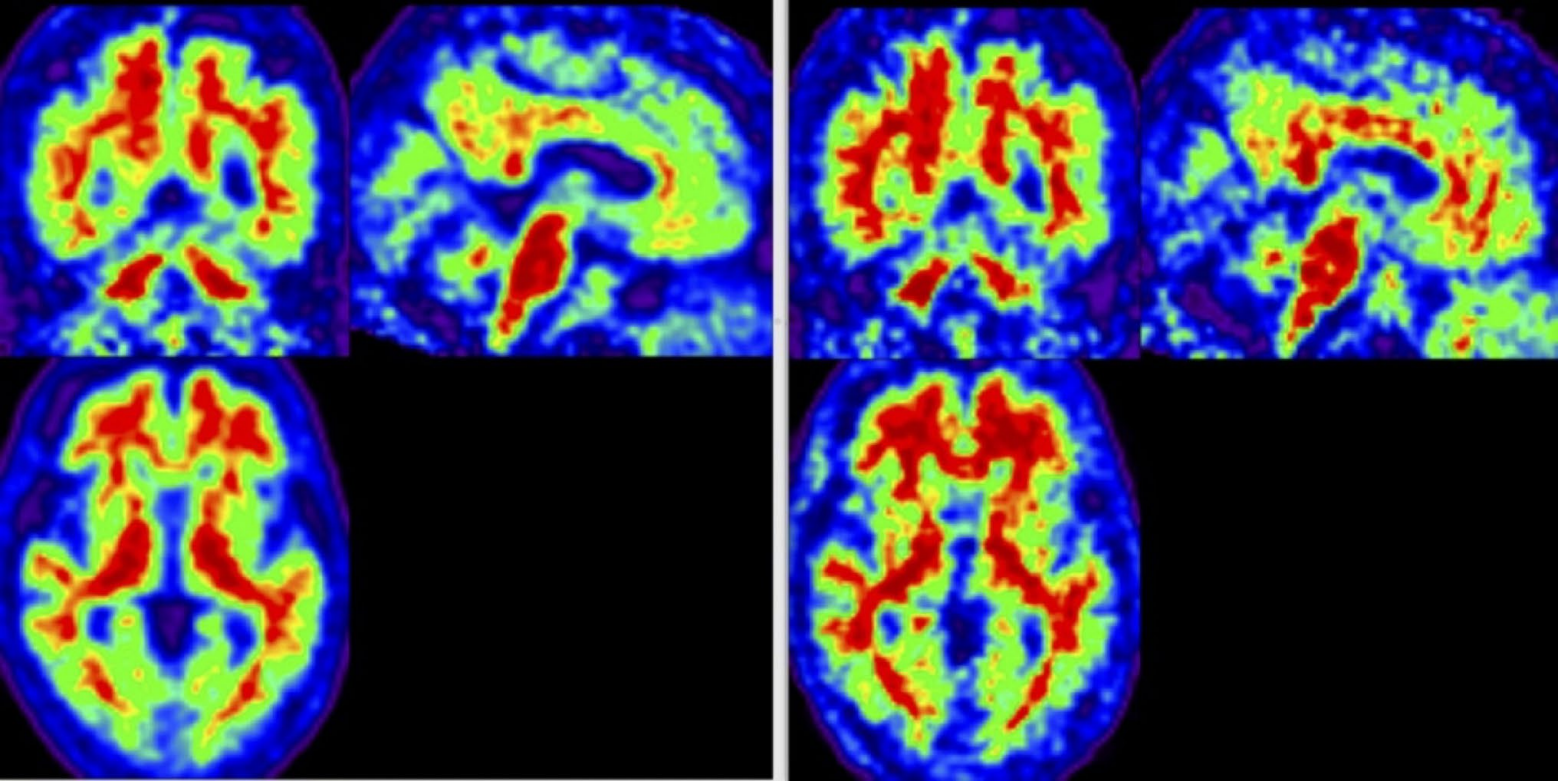
Representative ^18^F-flutemetamol PET images acquired using a dedicated brain PET scanner (right) and a conventional whole-body PET scanner (left). In the same AD case with focal amyloid deposition, the difference in spatial resolution between the two images is clearly evident. On the dedicated brain PET images, gray matter uptake in the left medial frontal lobe and in the bilateral posterior cingulate/precuneus is more clearly visualized

In recent years, several dedicated brain PET systems (Fig. 6) have been developed and are increasingly utilized in both research and clinical practice [33–35]. These systems offer superior spatial resolution, facilitating the detection of subtle amyloid and tau deposition at earlier stages of disease (Fig. 7).

Additionally, artificial intelligence (AI)–based image analysis is increasingly being applied to automate image interpretation, reduce inter-reader variability, and improve workflow efficiency in both clinical practice and clinical trials. For example, ultra-low-count ^18^F-FDG PET images in patients with dementia can now be reconstructed using deep learning (DL), enabling reductions in scan duration and administered activity without compromising diagnostic performance [36].

Deep learning–based methods have also been developed to predict amyloid deposition from FDG-PET [37–39]. Furthermore, DL-based approaches for FDG-PET interpretation [40, 41] and automated reading of amyloid PET [42] are expected to be useful in clinical practice. However, the final diagnosis should ultimately be made by a physician. Although promising, these models require external validation and standardization before routine clinical implementation.

## Future perspectives

The integration of amyloid PET, tau PET, quantitative biomarkers, and advanced image analysis will enable a more refined, precision-based approach to dementia care. As disease-modifying therapies continue to evolve, brain PET imaging is expected to remain central to individualized treatment strategies.

## Conclusion

In the era of anti-Aβ antibody therapy, brain PET imaging has transitioned from an adjunctive diagnostic tool to a cornerstone of clinical decision-making. Amyloid PET is indispensable for determining treatment eligibility and monitoring therapeutic response, whereas tau PET provides critical insights into disease stage and prognosis. Ongoing technological advances and standardization are expected to further enhance the role of PET imaging in precision medicine for dementia.
